# Use of High-Risk Medications Among Older Adults Enrolled in Medicare Advantage Plans vs Traditional Medicare

**DOI:** 10.1001/jamanetworkopen.2023.20583

**Published:** 2023-06-27

**Authors:** Jose F. Figueroa, Dannie Dai, Yevgeniy Feyman, Melissa M. Garrido, Thomas C. Tsai, E. John Orav, Austin B. Frakt

**Affiliations:** 1 Department of Health Policy and Management, Harvard T.H. Chan School of Public Health, Boston, Massachusetts; 2 Department of Medicine, Brigham and Women’s Hospital, Boston, Massachusetts; 3 Boston University School of Public Health, Boston, Massachusetts; 4 Veterans Affairs Boston Healthcare System, Boston, Massachusetts; 5 Department of Surgery, Brigham and Women’s Hospital, Boston, Massachusetts; 6 Center for Surgery and Public Health, Boston, Massachusetts

## Abstract

**Importance:**

Limiting the use of high-risk medications (HRMs) among older adults is a national priority to provide a high quality of care for older beneficiaries of both Medicare Advantage and traditional fee-for-service Medicare Part D plans.

**Objective:**

To evaluate the differences in the rate of HRM prescription fills among beneficiaries of traditional Medicare vs Medicare Advantage Part D plans and to examine the extent to which these differences change over time and the patient-level factors associated with higher rates of HRMs.

**Design, Setting, and Participants:**

This cohort study used a 20% sample of Medicare Part D data on filled drug prescriptions from 2013 to 2017 and a 40% sample from 2018. The sample comprised Medicare beneficiaries aged 66 years or older who were enrolled in Medicare Advantage or traditional Medicare Part D plans. Data were analyzed between April 1, 2022, and April 15, 2023.

**Main Outcomes and Measures:**

The primary outcome was the number of unique HRMs prescribed to older Medicare beneficiaries per 1000 beneficiaries. Linear regression models were used to model the primary outcome, adjusting for patient characteristics and county characteristics and including hospital referral region fixed effects.

**Results:**

The sample included 5 595 361 unique Medicare Advantage beneficiaries who were propensity score–matched on a year-by-year basis to 6 578 126 unique traditional Medicare beneficiaries between 2013 and 2018, resulting in 13 704 348 matched pairs of beneficiary-years. The traditional Medicare vs Medicare Advantage cohorts were similar in age (mean [SD] age, 75.65 [7.53] years vs 75.60 [7.38] years), proportion of males (8 127 261 [59.3%] vs 8 137 834 [59.4%]; standardized mean difference [SMD] = 0.002), and predominant race and ethnicity (77.1% vs 77.4% non-Hispanic White; SMD = 0.05). On average in 2013, Medicare Advantage beneficiaries filled 135.1 (95% CI, 128.4-142.6) unique HRMs per 1000 beneficiaries compared with 165.6 (95% CI, 158.1-172.3) HRMs per 1000 beneficiaries for traditional Medicare. In 2018, the rate of HRMs had decreased to 41.5 (95% CI, 38.2-44.2) HRMs per 1000 beneficiaries in Medicare Advantage and to 56.9 (95% CI, 54.1-60.1) HRMs per 1000 beneficiaries in traditional Medicare. Across the study period, Medicare Advantage beneficiaries received 24.3 (95% CI, 20.2-28.3) fewer HRMs per 1000 beneficiaries per year compared with traditional Medicare beneficiaries. Female, American Indian or Alaska Native, and White populations were more likely to receive HRMs than other groups.

**Conclusion and Relevance:**

Results of this study showed that HRM rates were consistently lower among Medicare Advantage than traditional Medicare beneficiaries. Higher use of HRMs among female, American Indian or Alaska Native, and White populations is a concerning disparity that requires further attention.

## Key Points

**Question:**

Are there meaningful differences in the use of high-risk medications (HRMs) among older Medicare beneficiaries enrolled in Medicare Advantage vs traditional fee-for-service Medicare Part D plans?

**Findings:**

In this cohort study of 13 704 348 matched pairs of beneficiary-years, lower adjusted rates of filled HRMs were found among those enrolled in Medicare Advantage plans compared with traditional Medicare, and the difference in rates between the 2 insurance types narrowed between 2013 and 2018. Female, American Indian or Alaska Native, and non-Hispanic White populations had the highest use of HRMs compared with other minority groups.

**Meaning:**

Findings of this study suggest an association between enrollment in Medicare Advantage plans and lower rates of filled HRMs among older Medicare beneficiaries, although concerning disparities exist, particularly in female, American Indian or Alaska Native, and White populations.

## Teaser

This cohort study evaluates the rate of high-risk medication prescription fills, changes over time, and patient-level factors associated with these rates among older adults with traditional Medicare vs Medicare Advantage Part D plans.

## Introduction

Achieving a high quality of care for older adults (66 years or older) is a national priority, 1 domain of which is safe prescribing practices.^1^ High-risk medications (HRMs) are defined as medications that should be avoided for older patients because of age-related changes in pharmacodynamics and chronic illness burden that may increase these patients’ risk of avoidable hospitalization, health care spending, and death.^2,3,4^ Despite consensus that HRMs should be minimized, they are commonly prescribed to older adults.^5^ As people live longer and multimorbidity continues to rise,^6^ the risk of potentially harmful drug–disease interactions and adverse events will also likely increase. Therefore, identification of strategies that reduce the use of HRMs in this population is critically important.

Since 2007, the quality of care of Medicare Advantage Part D plans (hereafter, Medicare Advantage), an alternative private coverage to traditional, stand-alone fee-for-service Medicare Part D plans (hereafter, traditional Medicare), has been monitored using quality measures from the Healthcare Effectiveness Data and Information Set (HEDIS).^7^ Specifically, Medicare Advantage plans are mandated to report their HRM rates under the DAE measure (Use of High-Risk Medications in Older Adults).^8^ The DAE measure defines an HRM using a list of drugs that should not be prescribed to Medicare beneficiaries aged 66 years or older.^9,10,11,12,13^

Efforts to curb HRMs have increased over the past decade through promotion of alternative medications, prior authorization, and geriatric consultation.^2,14^ There are reasons to believe that Medicare Advantage plans might have better management of HRM rates given their more aggressive utilization management strategies, whereby they contract with clinicians who generally perform better on quality measures.^15,16,17^ Additionally, given that Medicare Advantage plans are at risk for total costs of care,^18^ including costs of complications associated with HRM use, they may have more incentive to minimize HRMs. While traditional Medicare does not have direct national incentives for clinicians to improve their performance on HEDIS measures, more recent efforts such as the Merit-based Incentive Payment System,^19^ which includes the DAE measure as an optional quality measure for clinicians to report, may similarly be changing prescription patterns over time.

To date, however, it is not clear how rates of HRMs vary between Medicare Advantage and traditional Medicare beneficiaries despite national attention on differences in quality of care between Medicare Advantage and traditional Medicare.^20^ There is a small body of literature using data from 2013 that showed declining rates of HRMs and characterized the rate of HRMs in different patient subgroups.^5,21,22,23,24^ However, there have been no studies using more contemporary data at a national level that evaluate rates of HRMs by Medicare insurance over time. As the number of beneficiaries enrolling in Medicare Advantage has continued to increase, from 17% in 2000 to 48% in 2022, understanding the extent to which HRM prescription patterns differ by Medicare insurance type has become essential to inform existing and new medication management policies, public reporting quality measures, and financial incentives.^25^

Therefore, using national Medicare Part D data, we sought to answer several key questions. First, are there meaningful differences in the rate of HRM prescription fills among beneficiaries of traditional Medicare vs Medicare Advantage Part D plans? If so, to what extent do these differences change over time? Are there important patient-level factors associated with higher rates of HRMs?

## Methods

This cohort study was approved by the institutional review board at the Harvard T.H. Chan School of Public Health, which waived the informed consent requirement because of the inability to contact enrollees included in national deidentified claims data. We followed the Strengthening the Reporting of Observational Studies in Epidemiology (STROBE) reporting guideline.

### Data

We used a 20% sample of Medicare Part D data on filled drug prescriptions from 2013 to 2017 and a 40% sample from 2018 (based on research-identifiable data that were available to our research group). We used the base Medicare Master Beneficiary Summary File to obtain demographic data, including age, sex, race and ethnicity, dual-eligibility status for Medicaid, and county of residence. We limited the sample to beneficiaries aged 66 years or older as defined by the HEDIS DAE measure.^9,10,11,12,13^ We used Social Vulnerability Index (SVI) data from 2018.^26^ We categorized counties as rural or not rural using definitions from the US Office of Management and Budget.^27^ We used the Medicare Part D Master Beneficiary Summary File to determine eligibility for Medicare’s low-income subsidy (LIS), which helps beneficiaries pay for prescription drug coverage and costs.

Additionally, we obtained from the 2013 to 2018 HEDIS DAE measures the lists of drug products and National Drug Codes (NDCs) that were flagged as potentially harmful if prescribed to older adults.^9,10,11,12,13^ Because the NDCs list change year to year, for the main analyses we obtained a list of NDCs that were present each year of the 6-year study period (2013 to 2018), none of which had days’ supply criteria (eFigures 1 and 2 in the Supplement 1). As a secondary analysis, we repeated the main model using all NDCs that were flagged in each year of the study period. Nondrug prescriptions (eg, syringes and sponges) were excluded from analyses.

### Variables

All analyses were conducted at the beneficiary-year level. First, to identify unique HRMs, we collapsed NDCs of the same generic name into 1 entry for a given beneficiary-year. If any NDC under a generic name was classified as high risk, the entry for the generic name was considered as an HRM. The primary outcome was the number of unique HRMs prescribed to older adults. This outcome allowed for an estimation of the number of unique HRMs prescribed to older Medicare beneficiaries per 1000 beneficiaries. The secondary outcome was the proportion of older beneficiaries who received at least 1 HRM per year. This outcome allowed for the calculation of a binary measure of whether an older beneficiary received 1 or more HRM each year. Other secondary outcomes were the proportion of older beneficiaries who received at least 2 HRMs per year or the same HRM twice within a given year.

The primary variable of interest was Medicare insurance type (Medicare Advantage vs traditional Medicare). Covariates included race and ethnicity, age, sex, dual-eligibility status, LIS eligibility, SVI of county of residence, rurality of county of residence, and a proxy for patient severity defined as the number of unique drugs that were not considered HRMs. We used the latter measure in the absence of reliable and complete data.^28^ Race and ethnicity were defined using the Research Triangle Institute race code variable, which was based on a modified algorithm that uses self-reported data, name, and geographic location to group patients into the following categories: American Indian or Alaska Native, Asian or Pacific Islander, Black or African American, Hispanic, non-Hispanic White, other, and unknown.^29,30^ Race and ethnicity were analyzed given the well-documented inequities in access to high-quality health care among racial and ethnic minority groups that may increase their risk of receiving HRMs.

### Statistical Analyses

For each Medicare Advantage beneficiary in each year, we first restricted the traditional Medicare beneficiary controls to those residing in the same hospital referral region (HRR) in the same year. We used propensity score matching to identify the nearest-neighbor traditional Medicare beneficiary match (1:1) based on the covariates and a caliper of 0.2. We also compared demographic characteristics between the matched Medicare Advantage and traditional Medicare beneficiaries across the study years using standardized mean difference (SMD); an SMD less than 0.10 is generally considered negligible.^31^

Next, we used linear regressions to model the primary outcome, adjusting for the covariates, and the secondary outcomes, including indicator variables for the calendar year, fixed effects for HRRs, and plan-level random effects. We plotted the adjusted rate of unique HRMs prescribed per beneficiary in Medicare Advantage vs traditional Medicare in each year. We also plotted the adjusted proportion of older beneficiaries who received at least 1 HRM per year by Medicare insurance type. We estimated HRM rates over time across race and ethnicity, stratified by insurance type. Additionally, we reran the main model stratified by HRM drug class type.

Propensity score matching was conducted using R, version 4.2.1 (R Foundation for Statistical Computing). All regression analyses were performed using Stata/MP 16.1 (StataCorp LLC), with 2-tailed *t* tests, where applicable, and a *P* = .05 to establish statistical significance. Data were analyzed between April 1, 2022, and April 15, 2023.

Additionally, we conducted sensitivity analyses. First, we repeated the primary outcome models but expanded the definition of an HRM to include any high-risk NDCs that were flagged by HEDIS across the study period. Second, we confirmed the model results using a quasi-Poisson regression for the primary outcomes and a logistic regression model for the secondary outcomes. Third, we repeated the secondary outcomes model using 2 versions of the secondary HEDIS DAE measures: whether a beneficiary received at least 2 different HRMs and whether a beneficiary received the same HRM twice in a year. Fourth, we reran the main model and limited it to beneficiaries who were alive for 12 months of a year. Fifth, we reran the main model and used county rather than HRR fixed effects. Sixth, we ran models that directly adjusted for patient severity using clinical Hierarchical Condition Category (HCC) risk scores^32^ rather than the number of non-HRMs; this analysis was conducted using a 20% sample of 2018 Medicare Advantage Encounter data (based on availability to our research group). Within this analysis, we accounted for potential Medicare Advantage upcoding based on range estimates in the current literature by deflating Medicare Advantage beneficiary HCC risk scores by 6%, 11%, and 16%.^33,34^ Seventh, we reran the main analyses on the full sample of Medicare Advantage and traditional Medicare beneficiaries without the use of propensity score matching to evaluate whether results were consistent and for better generalizability.

## Results

Across the study period, the sample included 5 595 361 unique Medicare Advantage beneficiaries who were propensity score–matched on a year-by-year basis to 6 578 126 unique traditional Medicare beneficiaries between 2013 and 2018, resulting in 13 704 348 matched pairs of beneficiary-years (Table). The traditional Medicare beneficiaries had a mean (SD) age of 75.65 (7.53) years. The sample comprised 5 577 087 females (40.7%) and 8 127 261 males (59.3%) and included individuals with American Indian or Alaska Native (0.2%), Asian (4.3%), Black (8.9%), Hispanic (7.5%), White (77.1%), or other race and ethnicity (2.1%). The Medicare Advantage beneficiaries had a mean (SD) age of 75.60 (7.38) years. The sample comprised 5 566 514 females (40.6%) and 8 137 834 males (59.4%) and included individuals with American Indian or Alaska Native (0.2%), Asian (3.7%), Black (10.0%), Hispanic (6.6%), White (77.4%), or other race and ethnicity (2.1%). Among these 2 groups, 16.6% vs 15.5% had dual-eligibility status, and 12.0% vs 11.7% lived in rural areas (SMD, <0.10) (Table). The characteristics of the full sample are shown in eTable 1 in Supplement 1.

**Table.** Characteristics of the Matched Sample of Beneficiaries in Medicare Advantage vs Traditional Medicare From 2013 to 2018^a^

Patient characteristic	Traditional Medicare, No. (%)	Medicare Advantage, No. (%)	SMD^b^
All beneficiary-years	13 704 348	13 704 348	NA
Age group, y			
66-70	4 216 776 (30.8)	4 194 235 (30.6)	0.05
71-75	3 610 047 (26.3)	3 642 226 (26.6)	
76-80	2 469 140 (18.0)	2 493 116 (18.2)	
81-90	2 796 136 (20.4)	2 780 241 (20.3)	
≥90	612 249 (4.5)	594 530 (4.3)	
Sex			
Male	8 127 261 (59.3)	8 137 834 (59.4)	0.002
Female	5 577 087 (40.7)	5 566 514 (40.6)	
Race and ethnicity^c^			
American Indian or Alaska Native	25 544 (0.2)	28 645 (0.2)	0.05
Asian or Pacific Islander	586 004 (4.3)	510 562 (3.7)	
Black or African American	1 221 003 (8.9)	1 365 325 (10.0)	
Hispanic	1 023 843 (7.5)	909 316 (6.6)	
Non-Hispanic White	10 566 269 (77.1)	10 605 693 (77.4)	
Other^d^	281 685 (2.1)	284 807 (2.1)	
Dual-eligibility status			
No	11 427 948 (83.4)	11 574 972 (84.5)	0.03
Yes	2 276 400 (16.6)	2 129 376 (15.5)	
LIS eligibility			
No	10 843 761 (79.1)	10 942 396 (79.8)	0.02
Yes	2 860 587 (20.9)	2 761 952 (20.2)	
Rurality of county of residence			
Not rural	12 062 653 (88.0)	12 105 433 (88.3)	0.01
Rural	1 641 695 (12.0)	1 598 915 (11.7)	
Neighborhood deprivation			
SVI of county of residence	0.38 (11.58)	0.36 (12.50)	0.002

Abbreviations: LIS, low-income subsidy; NA, not applicable; SMD, standardized mean difference; SVI, Social Vulnerability Index.

^a^ Across the study period, the sample included 5 595 361 unique Medicare Advantage beneficiaries matched on a year-by-year basis to 6 578 126 unique traditional Medicare beneficiaries between 2013 and 2018, resulting in 13 704 348 matched pairs of beneficiary-years.

^b^ An SMD less than 0.10 was considered negligible.

^c^ Race and ethnicity were obtained from the Medicare Master Beneficiary Summary File and defined using the Research Triangle Institute race code variable.

^d^ Other category was not specified.

### Use of HRM by Medicare Insurance Type

There were 3134 unique HRMs that were flagged consistently during all study years in the HEDIS DAE measure, which were considered in the primary analyses (eFigure 1 in Supplement 1). The drug categories represented by the unique HRMs were similar in proportion to the drug categories, including all flagged HRMs, within a given year (eFigure 2 in Supplement 1).

**Figure 1.**
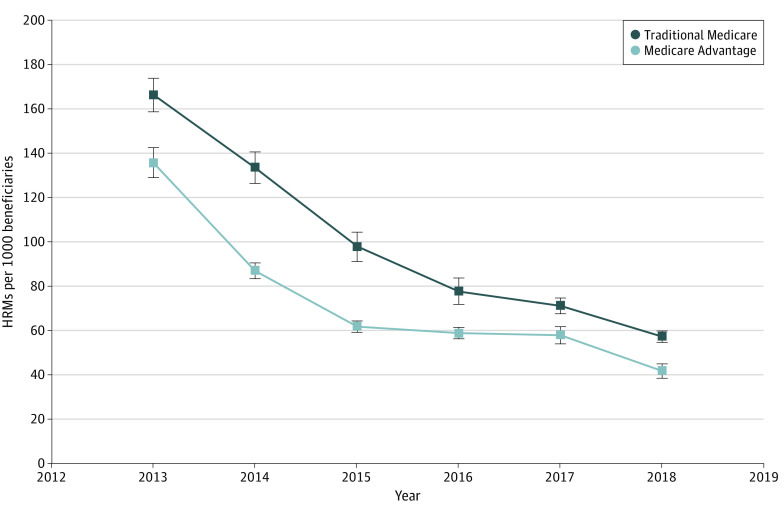
Adjusted Rate of High-Risk Medications (HRMs) per 1000 Medicare Beneficiaries in Medicare Advantage vs Traditional Medicare From 2013 to 2018 Error bars represent 95% CIs.

On average in 2013, Medicare Advantage beneficiaries were prescribed 135.1 (95% CI, 128.4-142.6) unique HRMs per 1000 beneficiaries compared with 165.6 (95% CI, 158.1-172.3) HRMs per 1000 beneficiaries for traditional Medicare. Each year of the study period, the rate of HRMs decreased for traditional Medicare and Medicare Advantage. The gap between the 2 types narrowed over time. In 2018, the number of HRMs decreased to 41.5 (95% CI, 38.2-44.2) per 1000 beneficiaries in Medicare Advantage and to 56.9 (95% CI, 54.1-60.1) HRMs per 1000 beneficiaries in traditional Medicare (Figure 1). Similar patterns of HRM use were observed across all racial and ethnic groups in Medicare Advantage and traditional Medicare (eFigure 3 in Supplement 1). Across all drug categories, Medicare Advantage had lower HRM rates than traditional Medicare (eTable 2 and eFigure 4 in Supplement 1).

**Figure 2.**
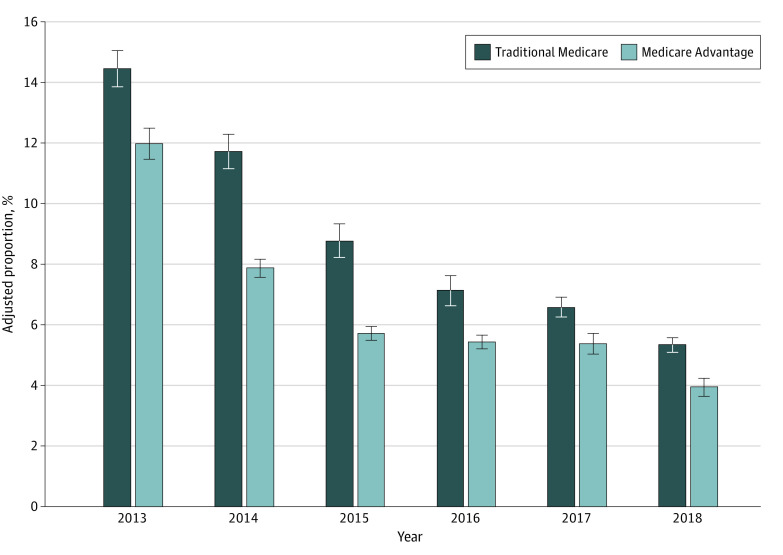
Adjusted Proportion of Beneficiaries in Medicare Advantage vs Traditional Medicare With at Least 1 High-Risk Medication From 2013 to 2018 Error bars represent 95% CIs.

Similar patterns were observed in the adjusted proportion of beneficiaries who received at least 1 HRM within a given year (Figure 2). In 2013, 11.9% (95% CI, 11.4%-12.5%) of Medicare Advantage beneficiaries received at least 1 HRM compared with 14.4% (95% CI, 13.8%-15.0%) of traditional Medicare beneficiaries. By 2018, the gap narrowed, but Medicare Advantage beneficiaries were still less likely to receive at least 1 HRM, with an adjusted proportion of 3.9% (95% CI, 3.6%-4.2%) vs 5.3% (95% CI, 5.1%-5.6%) for traditional Medicare beneficiaries.

### Association Between Medicare Insurance Type and Rates of Filled HRMs

**Figure 3.**
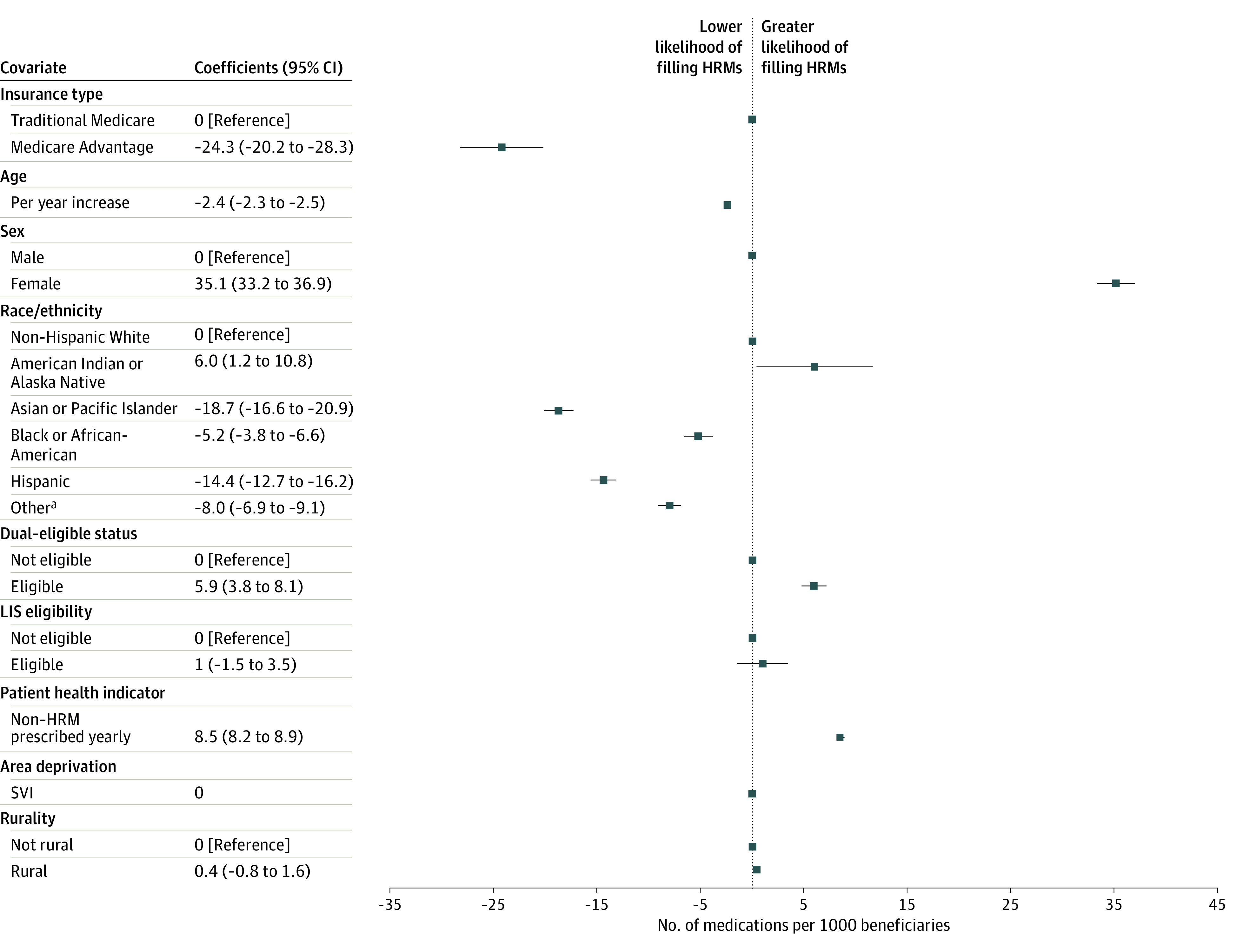
Association Between Medicare Insurance Type and Filled High-Risk Medication (HRM) Prescriptions From 2013 to 2018 Model also included year and hospital referral region fixed effects to control for market characteristics where patients reside. LIS indicates low-income subsidy; SVI, Social Vulnerability Index. ^a^Other category was not specified.

We compared the adjusted differences in the rates of HRMs across different patient characteristics. Compared with enrollment in traditional Medicare, enrollment in Medicare Advantage was associated with a lower likelihood of receiving HRMs. Beneficiaries in Medicare Advantage received 24.3 (95% CI, 20.2-28.3) fewer HRMs per 1000 beneficiaries compared with traditional Medicare beneficiaries (Figure 3). A 1-year increase in age was associated with 2.4 (95% CI, 2.3-2.5) fewer HRMs prescribed per 1000 beneficiaries. Dual-eligibility status was associated with 5.9 (95% CI, 3.8-8.1) more HRMs per 1000 beneficiaries compared with their noneligible counterparts. Reported female sex was associated with 35.1 (95% CI, 33.2-36.9) more HRMs than male sex. There were no associations between the rurality and SVI of county of residence or LIS eligibility and the rate of HRMs per 1000 beneficiaries.

We observed differences by race and ethnicity. Compared with White beneficiaries, Asian, Black, and Hispanic beneficiaries and those with other race and ethnicity were all less likely to receive HRMs. However, American Indian or Alaska Native beneficiaries received 6.0 (95% CI, 1.2-10.8) more HRMs compared with White beneficiaries (Figure 3). In the analyses of the secondary outcome, the association between patient characteristics and the secondary outcome was similar to the association with the primary outcome (eFigure 5 in Supplement 1). Among Medicare Advantage beneficiaries, those enrolled in preferred provider organization (PPO) plans received 9.4 (95% CI, 4.4-14.5) more HRMs than those enrolled in health maintenance organization (HMO) plans (eTable 3 in Supplement 1).

### Sensitivity Analyses

Across all sensitivity analyses, Medicare Advantage was consistently associated with a lower likelihood of receiving HRMs, including the analyses across all high-risk NDCs (eTable 4 in Supplement 1), when using quasi-Poisson regression and logistic regression (eTable 5 in Supplement 1), on secondary DAE measures (eTable 6 in Supplement 1), on beneficiaries who remained alive through 1 year (eTable 7 In Supplement 1), and in models using county rather than HRR fixed effects (eTable 8 in Supplement 1). The results remained consistent when repeating the analyses on the full sample without propensity score matching (eFigure 6 in Supplement 1). Additionally, the results remained consistent when directly adjusting for patient severity in place of non-HRMs using 2018 data (eTable 9 in Supplement 1).

## Discussion

In this national cohort study, prescribed HRMs received by older Medicare beneficiaries decreased from 2013 to 2018, continuing the improvement observed in prior data between 2007 to 2011.^24^ Rates of HRM use among Medicare Advantage beneficiaries was consistently lower than those in traditional Medicare, but the gap between these insurance types decreased over time. While the decline in HRM use across both traditional Medicare and Medicare Advantage is reassuring, higher rates of filled HRMs among the traditional Medicare, American Indian or Alaska Native, White, and female populations compared with their counterparts warrant further attention.

There are several factors that could help explain the lower rates of HRMs in Medicare Advantage. Prior work has documented that Medicare Advantage plans engage in aggressive utilization management strategies.^15,16,17,18^ It is possible that because Medicare Advantage plans are responsible for not only Part D care but also Parts A and B, their prescribing practices are more effective than those of traditional Medicare, minimizing subsequent cost of care.^18^ Additionally, Medicare Advantage’s capitation-based payment system incentivizes clinicians to avoid costly care.^15^ Medicare Advantage quality is directly incentivized through the Star Rating System and its associated bonus program to optimize performance on HEDIS quality-of-care measures given that they are rewarded for higher star ratings.^2,7,35,36,37,38,39,40^

We observed higher rates of filled HRMs in certain subgroups, including American Indian or Alaska Native and White populations. Given that HRM use was associated with worse clinical outcomes, these findings showed a potentially lower quality of care among clinicians who disproportionately served American Indian or Alaska Native beneficiaries, due in part to past and continued severe underfunding of health infrastructures for this population.^41^ As for higher rates among White populations vs other minority groups, some of these differences may be associated with underlying race-differential prescription bias from clinicians. For example, prior work has documented that clinicians were less likely to treat pain among Black than White populations for different medical and surgical conditions.^42,43,44,45^ Although we controlled for indicators of poverty, including dual eligibility for Medicaid, LIS status, and neighborhood vulnerability, it is possible that differences in patients’ ability to pay for medications might still affect the findings. Prior data have consistently shown that Black and Hispanic populations were less likely to be able to afford most medications, which could play a role in lower rates of filled HRMs.^46,47^

In addition, female patients were at a higher risk of filling HRMs. This finding was reported in previous literature and may be associated with the categorization of some oral estrogens as potentially high risk.^21,34^ However, further research is needed to understand how clinical settings and physician practice patterns may factor in the differences in prescribed HRMs by sex. We also found that Medicare Advantage beneficiaries enrolled in HMO plans had lower rates of HRMs than those enrolled in PPO plans. This finding is consistent with prior results showing lower use of health care services among those with HMO vs PPO plans.^48^

The study findings have important policy implications. The persistently higher rates of HRMs among the traditional Medicare population suggest that the Centers for Medicare & Medicaid Services (CMS) should consider more widespread incentivization of medication management to lower HRM rates to Medicare Advantage levels. One such strategy is including HRM rates as a quality measure for Medicare accountable care organizations, the largest alternative payment model in traditional Medicare. Currently, while the Merit-based Incentive Payment System—the mandatory quality-reporting program for clinicians who do not participate in accountable care organizations^48^—included the DAE measure, not all clinicians selected to report this. Additionally, the finding that HRM rates have decreased in traditional Medicare on a similar trajectory as Medicare Advantage plans necessitates further research. It is possible that HRM rates in traditional Medicare improved alongside Medicare Advantage’s rates given prior reports suggesting more stringent use of prior authorization in traditional Medicare during the study period.^35,36^ These findings cannot be explained by CMS Medication Therapy Management programs given reported low engagement among traditional Medicare beneficiaries.^39^ More research is needed to understand the mechanisms and incentives behind this pattern. The finding that American Indian or Alaska Native beneficiaries were at a high risk of receiving HRMs is concerning and calls for close monitoring of and strategies for improving medication management among clinicians who serve a disproportionate number of American Indian or Alaska Native older adults. The CMS should consider working closely with the Indian Health Services to further investigate and implement strategies to reduce these concerning disparities.

### Limitations

This study has some limitations. First, the main analyses focused on drugs that were included in the DAE measure in every year of the study period. However, the results were consistent even when expanding to any drug listed as an HRM under the DAE measure. Second, assessing the clinical appropriateness of filled prescription drugs was not possible in this study due to data restrictions; however, the HEDIS DAE measure explicitly also does not account for clinical appropriateness. Third, while the issue of selection bias between Medicare Advantage and traditional Medicare has become less of a concern,^49,50,51^ without directly adjusting for patient severity, the findings could be associated with differences in beneficiary medication needs rather than plan practices. However, we conducted propensity score matching to find more comparable Medicare Advantage and traditional Medicare beneficiaries, and in the sensitivity analyses (where we further adjusted for patient’s HCC risk scores), we found patterns that were consistent with those in the main analyses even after accounting for potential issues of upcoding in the Medicare Advantage population.

Fourth, the study focused on Medicare Advantage as a single exposure. There was potential variation across the different Medicare Advantage payers as evidenced by Medicare Advantage beneficiaries in HMO plans receiving fewer HRMs than beneficiaries in PPO plans. Fifth, the analyses were restricted to Medicare Advantage and traditional Medicare Part D beneficiaries who filled a prescription.

## Conclusions

While the gap has narrowed over time, the use of HRMs among Medicare Advantage beneficiaries was lower than the use among traditional Medicare beneficiaries. This finding may be associated with unique financial and quality incentives in Medicare Advantage plans. More research is needed to elucidate the incentives and mechanisms that may play a role in decreased HRM use among the traditional Medicare population. Additionally, the findings showed higher use of HRMs among female, American Indian or Alaska Native, and White populations, a disparity that requires further attention.

## Notes

Supplement 1. **eFigure 1.** Drugs Flagged as High-Risk Medications for the Elderly by HEDIS From 2013-2018 **eFigure 2.** Classification of Drugs Flagged as High-Risk Medications by Year **eFigure 3.** HRM Rates in Across Race/Ethnicity Stratified by Medicare Insurance Type, 2013-2018 **eFigure 4.** Adjusted Yearly Rates of HRMs per 1,000 MA and TM Beneficiaries Stratified by HRMs Drug Category, 2013-2018 **eFigure 5.** Association Between Beneficiary Characteristics and Proportion That Received One or More HRMs **eFigure 6.** Association Between Medicare Insurance Type and Filled HRM Prescriptions Using Full Unmatched Sample of Medicare Beneficiaries, 2013-2018 **eTable 1.** Characteristics of Full Medicare Beneficiary Sample Before Propensity Score Matching, 2013-2018 **eTable 2.** HRM Rates in MA vs TM Beneficiaries Stratified by HRMs Drug Category, 2013-2018 **eTable 3.** HRM Rates Among Medicare Advantage PPO Compared to HMO Plans, 2013-2018 **eTable 4.** HRM Rates in MA vs TM Beneficiaries After Expanding Study Definition of HRM to Any NDCs Flagged Within a Given Year, 2013-2018 **eTable 5.** HRM Rates in MA vs TM Beneficiaries Using Negative Binomial Regressions and Logistic Regressions, 2013-2018 **eTable 6.** Differences in Secondary HEDIS DAE Measures Among MA vs TM Beneficiaries, 2013-2018 **eTable 7.** HRM Rates in MA vs TM Beneficiaries That Remained Alive for All 12 Months of a Year, 2013-2018 **eTable 8.** HRM Rates in MA vs TM Beneficiaries With County Rather Than HRR Fixed Effects, 2013-2018 **eTable 9.** HRM Rates in MA vs TM Beneficiaries Adjusting for Patient HCC Risk Score vs # of non-HRMs Received, 2018

Supplement 2.
Data Sharing Statement
