# Supplementary material for: Use of High-Risk Medications Among Older Adults Enrolled in Medicare Advantage Plans vs Traditional Medicare
Source: JAMA Netw Open. 2023 Jun 27;6(6):e2320583. doi: 10.1001/jamanetworkopen.2023.20583 (PMC10300714; doi:10.1001/jamanetworkopen.2023.20583)
Supplement: Supplement 1. — eFigure 1. Drugs Flagged as High-Risk Medications for the Elderly by HEDIS From 2013-2018 eFigure 2. Classification of Drugs Flagged as High-Risk Medications by Year eFigure 3. HRM Rates in Across Race/Ethnicity Stratified by Medicare Insurance Type, 2013-2018 eFigure 4. Adjusted Yearly Rates of HRMs per 1,000 MA and TM Beneficiaries Stratified by HRMs Drug Category, 2013-2018 eFigure 5. Association Between Beneficiary Characteristics and Proportion That Received One or More HRMs eFigure 6. Association Between Medicare Insurance Type and Filled HRM Prescriptions Using Full Unmatched Sample of Medicare Beneficiaries, 2013-2018 eTable 1. Characteristics of Full Medicare Beneficiary Sample Before Propensity Score Matching, 2013-2018 eTable 2. HRM Rates in MA vs TM Beneficiaries Stratified by HRMs Drug Category, 2013-2018 eTable 3. HRM Rates Among Medicare Advantage PPO Compared to HMO Plans, 2013-2018 eTable 4. HRM Rates in MA vs TM Beneficiaries After Expanding Study Definition of HRM to Any NDCs Flagged Within a Given Year, 2013-2018 eTable 5. HRM Rates in MA vs TM Beneficiaries Using Negative Binomial Regressions and Logistic Regressions, 2013-2018 eTable 6. Differences in Secondary HEDIS DAE Measures Among MA vs TM Beneficiaries, 2013-2018 eTable 7. HRM Rates in MA vs TM Beneficiaries That Remained Alive for All 12 Months of a Year, 2013-2018 eTable 8. HRM Rates in MA vs TM Beneficiaries With County Rather Than HRR Fixed Effects, 2013-2018 eTable 9. HRM Rates in MA vs TM Beneficiaries Adjusting for Patient HCC Risk Score vs # of non-HRMs Received, 2018 [file jamanetwopen-e2320583-s001.pdf]

Supplemental Online Content

Figueroa JF, Dai D, Feyman Y, et al. Use of high-risk medications among older adults enrolled in Medicare Advantage plans vs traditional Medicare. *JAMA Netw Open*. 2023;6(6):e2320583. doi:10.1001/jamanetworkopen.2023.20583

eFigure 1. Drugs Flagged as High-Risk Medications for the Elderly by HEDIS From 2013-2018

eFigure 2. Classification of Drugs Flagged as High-Risk Medications by Year

eFigure 3. HRM Rates in Across Race/Ethnicity Stratified by Medicare Insurance Type, 2013-2018

eFigure 4. Adjusted Yearly Rates of HRMs per 1,000 MA and TM Beneficiaries Stratified by HRMs Drug Category, 2013-2018

eFigure 5. Association Between Beneficiary Characteristics and Proportion That Received One or More HRMs

eFigure 6. Association Between Medicare Insurance Type and Filled HRM Prescriptions Using Full Unmatched Sample of Medicare Beneficiaries, 2013-2018

eTable 1. Characteristics of Full Medicare Beneficiary Sample Before Propensity Score Matching, 2013-2018

eTable 2. HRM Rates in MA vs. TM Beneficiaries Stratified by HRMs Drug Category, 2013-2018

eTable 3. HRM Rates Among Medicare Advantage PPO Compared to HMO Plans, 2013-2018

eTable 4. HRM Rates in MA vs. TM Beneficiaries After Expanding Study Definition of HRM to Any NDCs Flagged Within a Given Year, 2013-2018

eTable 5. HRM Rates in MA vs. TM Beneficiaries Using Negative Binomial Regressions and Logistic Regressions, 2013-2018

eTable 6. Differences in Secondary HEDIS DAE Measures Among MA vs. TM Beneficiaries, 2013-2018

eTable 7. HRM Rates in MA vs. TM Beneficiaries That Remained Alive for All 12 Months of a Year, 2013-2018

© 2023 Figueroa JF et al. *JAMA Network Open*

eTable 8. HRM Rates in MA vs. TM Beneficiaries With County Rather Than HRR Fixed Effects, 2013-2018

eTable 9. HRM Rates in MA vs. TM Beneficiaries Adjusting for Patient HCC Risk Score vs # of non-HRMs Received, 2018

This supplemental material has been provided by the authors to give readers additional information about their work.

© 2023 Figueroa JF et al. *JAMA Network Open*

eFigure 1: Drugs Flagged as High-Risk Medications for the Elderly by HEDIS from 2013-2018

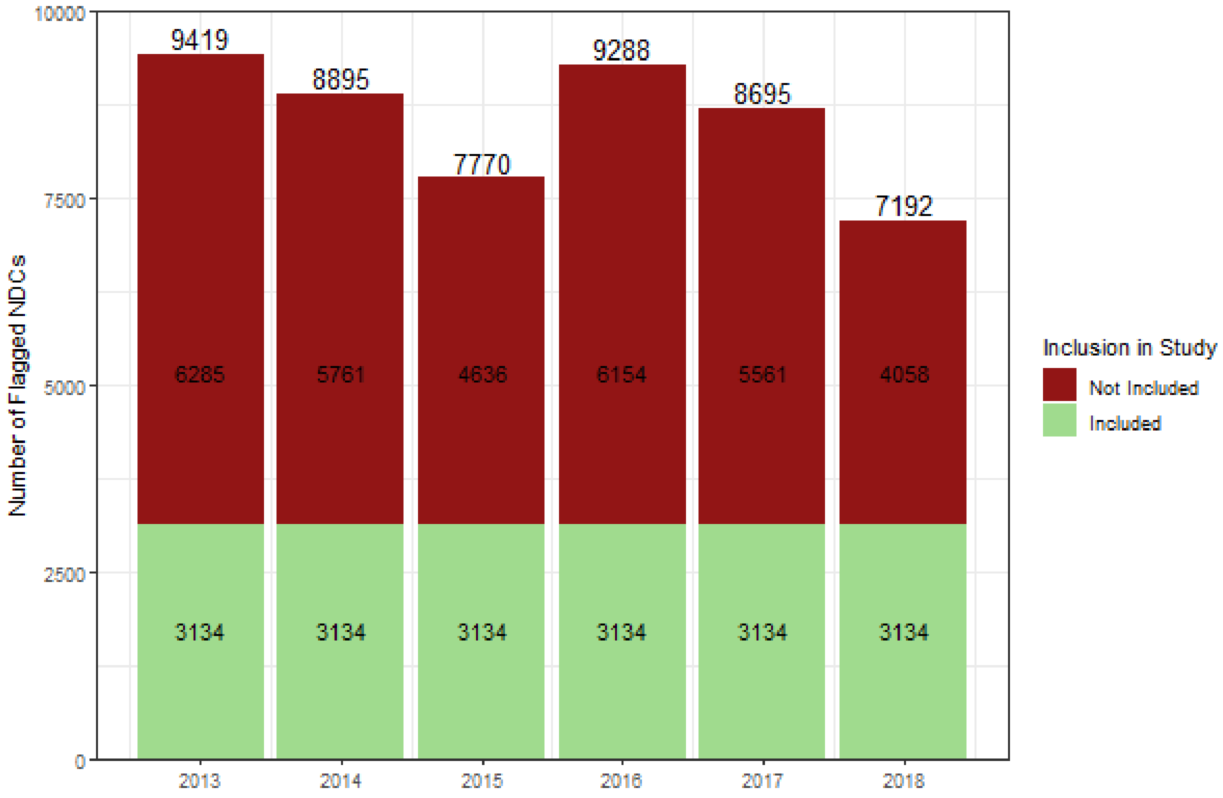

Caption: The 3,134 unique medications that were flagged across all years from 2013-2018 were considered in our primary outcome and analyses of this study.

© 2023 Figueroa JF et al. *JAMA Network Open*

eFigure 2: Classification of Drugs Flagged as High-Risk Medications by Year

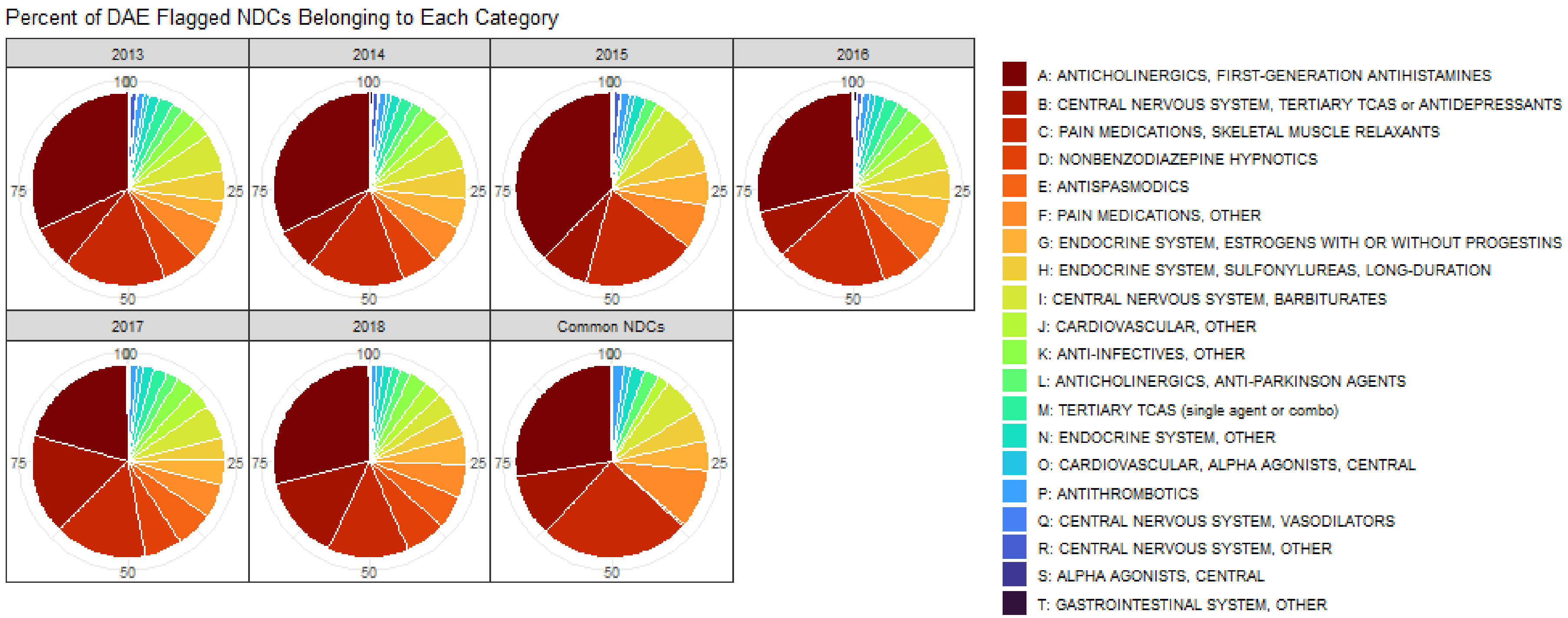

© 2023 Figueroa JF et al. *JAMA Network Open*

eFigure 3: HRM Rates in Across Race/Ethnicity Stratified by Medicare Insurance Type, 2013-2018

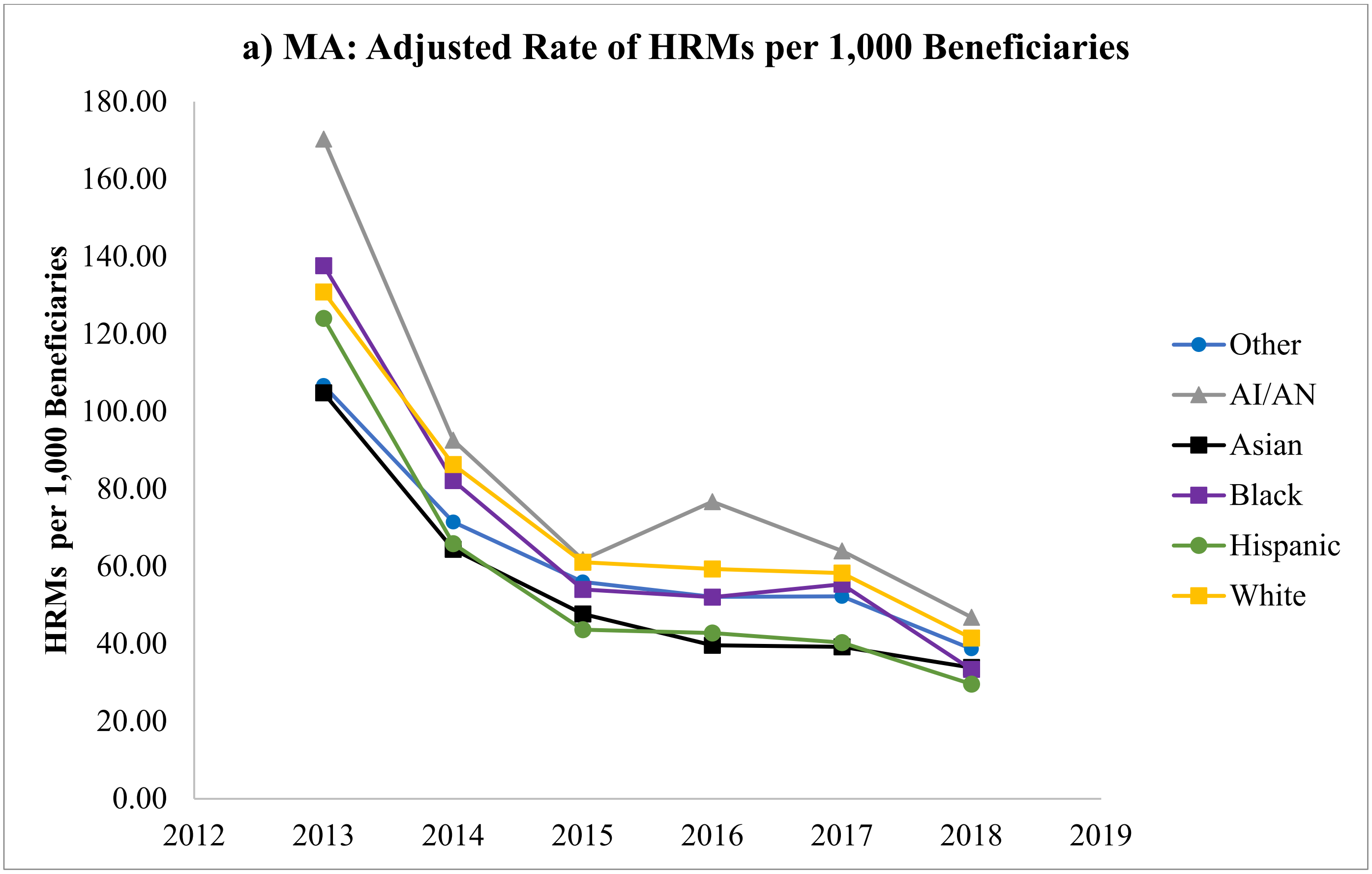

© 2023 Figueroa JF et al. *JAMA Network Open*

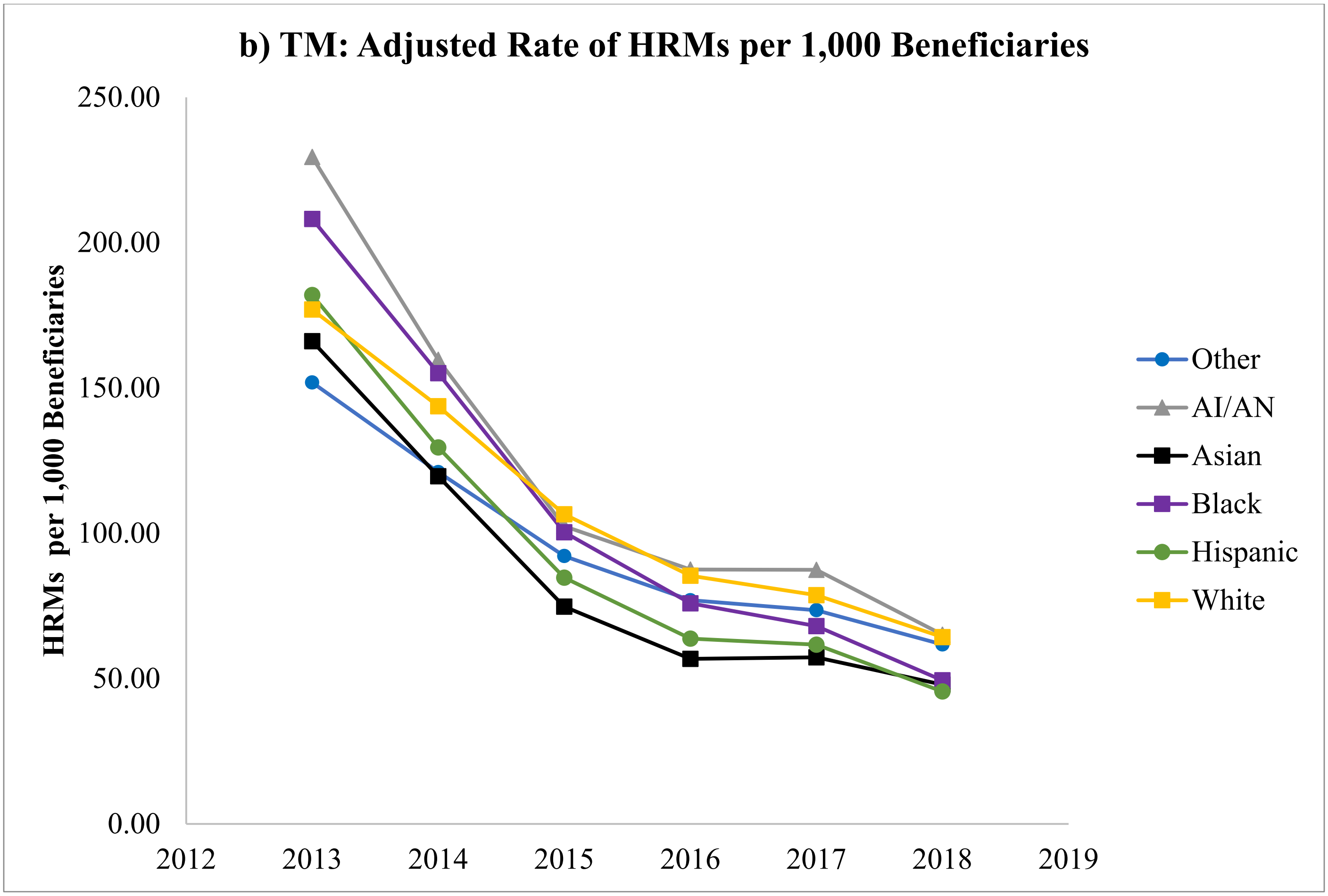

© 2023 Figueroa JF et al. *JAMA Network Open*

eFigure 4: Adjusted Yearly Rates of HRMs per 1,000 MA and TM Beneficiaries Stratified by HRMs Drug Category, 2013-2018

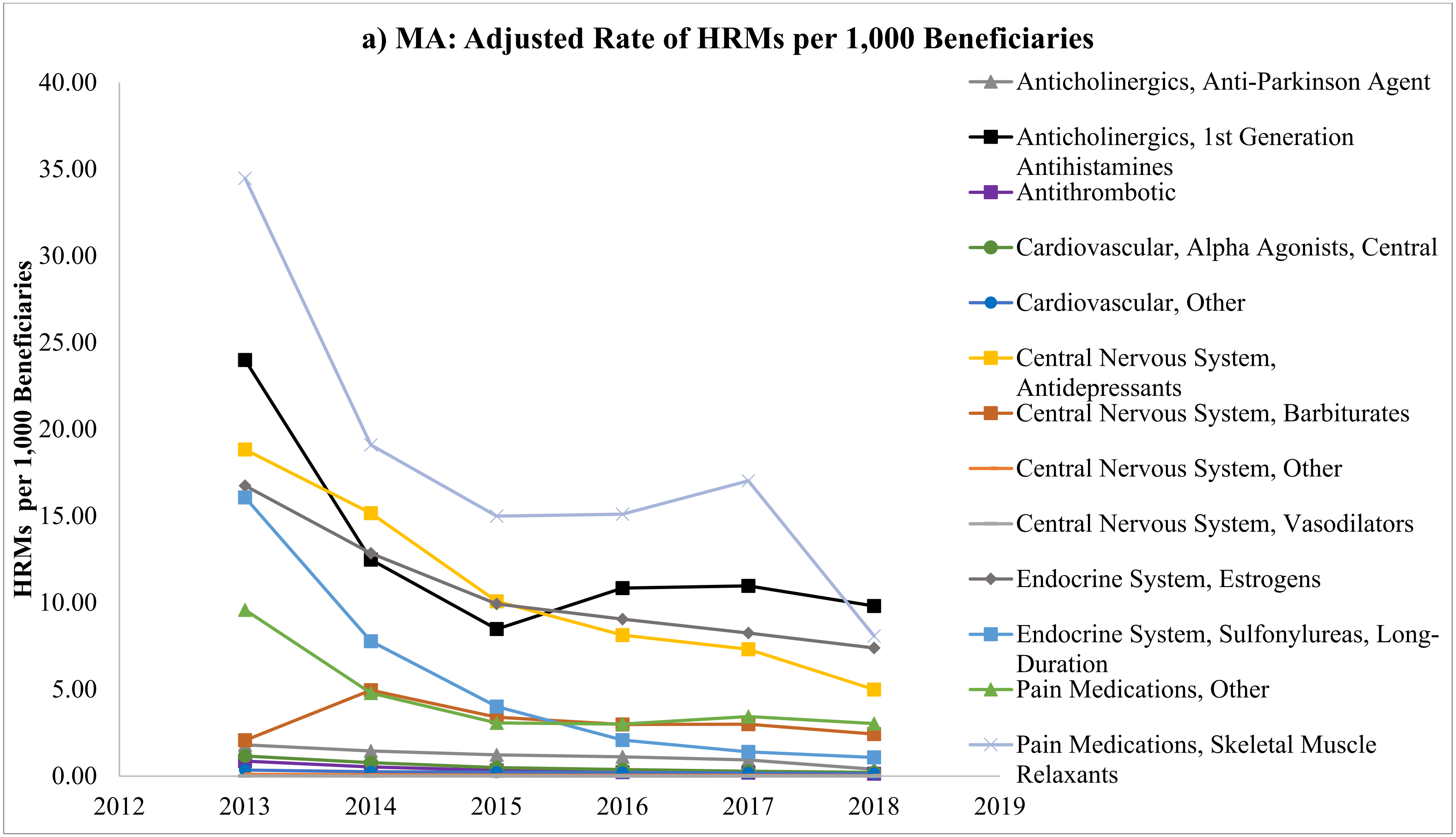

© 2023 Figueroa JF et al. *JAMA Network Open*

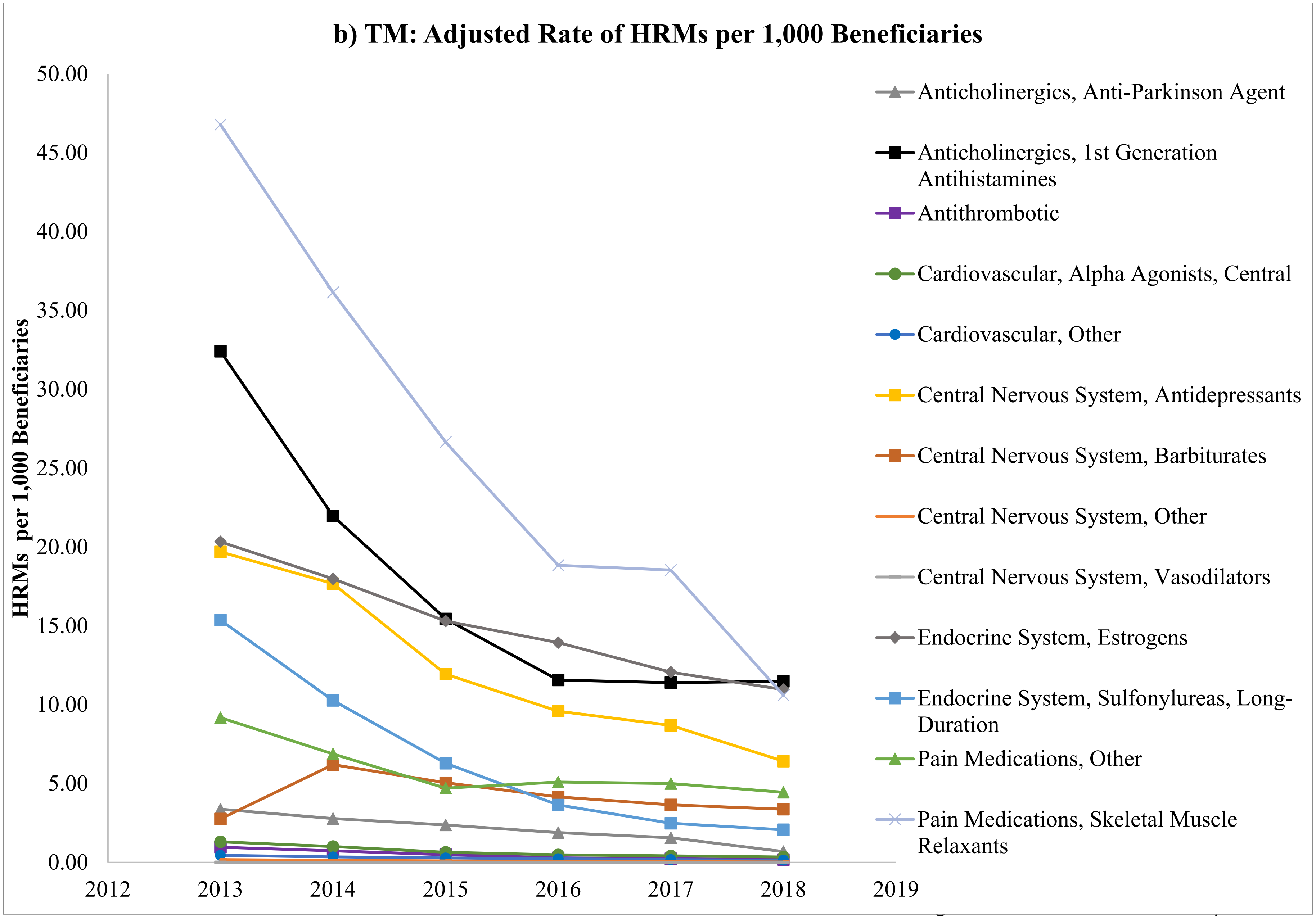

b) TM: Adjusted Rate of HRMs per 1,000 Beneficiaries
HRMs per 1,000 Beneficiaries
50.00
45.00
40.00
35.00
30.00
25.00
20.00
15.00
10.00
5.00
0.00
2012
2013
2014
2015
2016
2017
2018
2019
Anticholinergics, Anti-Parkinson Agent
Anticholinergics, 1st Generation Antihistamines
Antithrombotic
Cardiovascular, Alpha Agonists, Central
Cardiovascular, Other
Central Nervous System, Antidepressants
Central Nervous System, Barbiturates
Central Nervous System, Other
Central Nervous System, Vasodilators
Endocrine System, Estrogens
Endocrine System, Sulfonylureas, Long-Duration
Pain Medications, Other
Pain Medications, Skeletal Muscle Relaxants

eFigure 5: Association Between Beneficiary Characteristics and Proportion that Received One or More HRMs

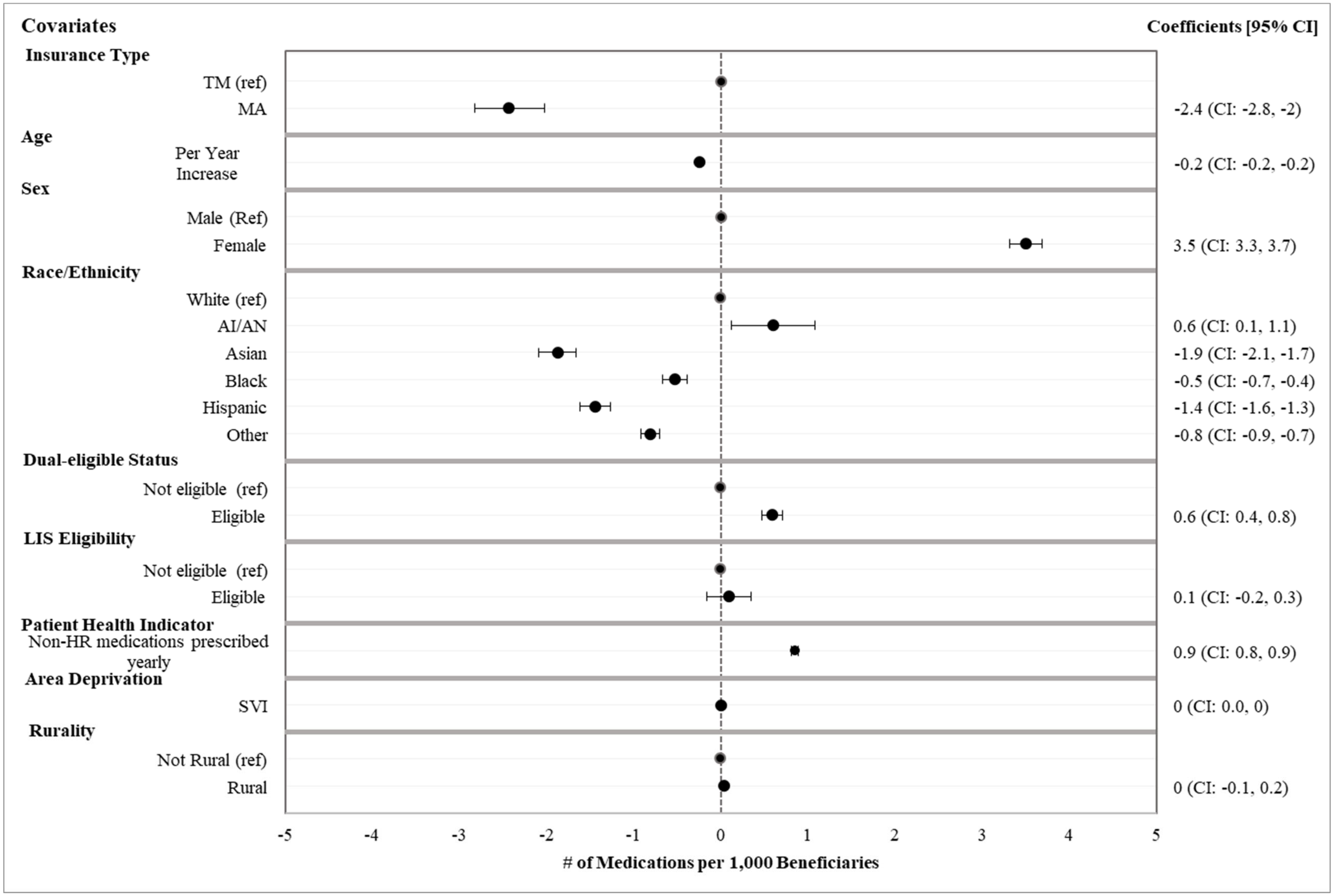

© 2023 Figueroa JF et al. *JAMA Network Open*

eFigure 6: Association Between Medicare Insurance Type and Filled HRM Prescriptions Using Full Unmatched Sample of Medicare Beneficiaries, 2013-2018

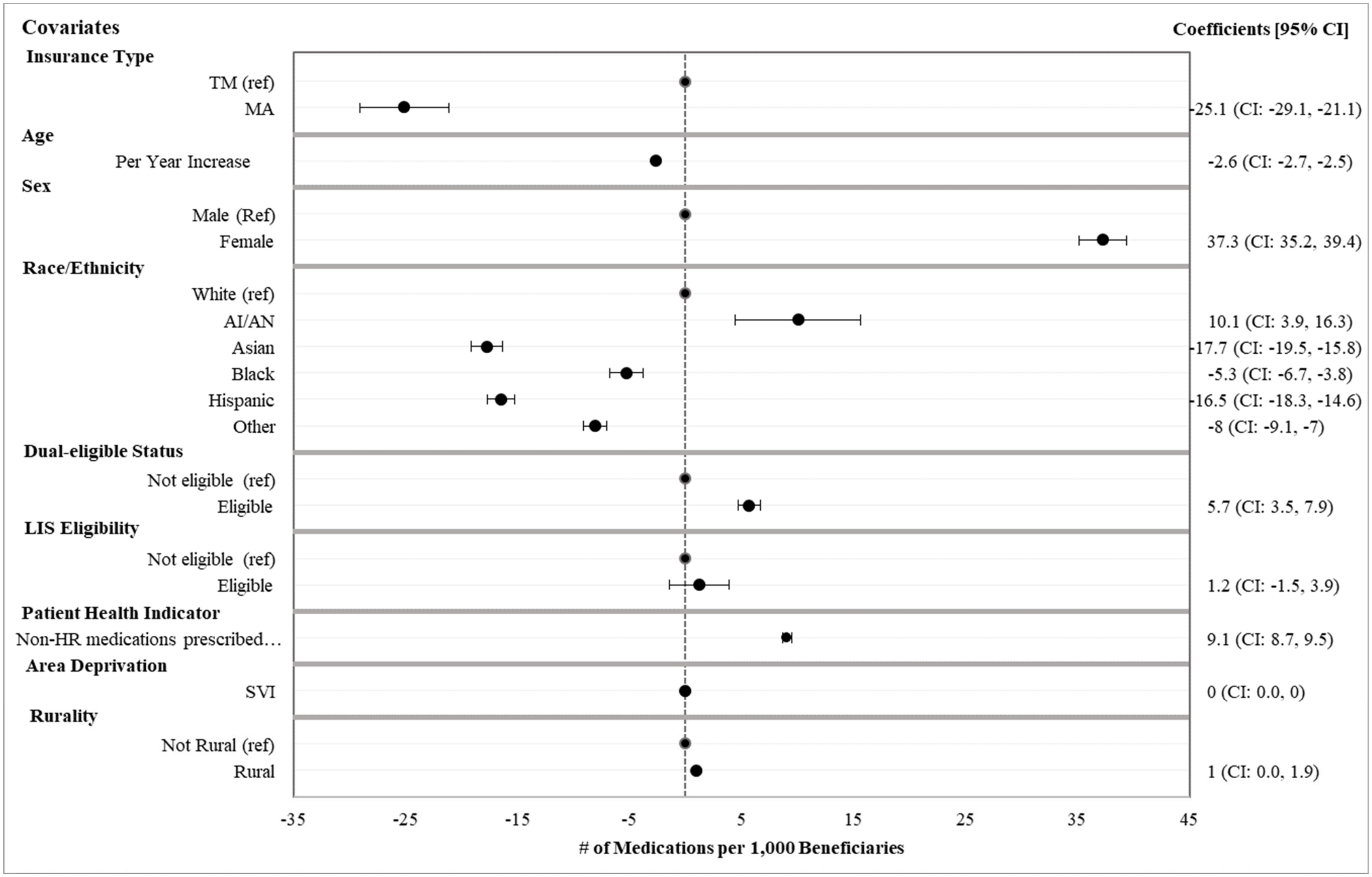

© 2023 Figueroa JF et al. *JAMA Network Open*

eTable 1: Characteristics of Full Medicare Beneficiary Sample before Propensity Score Matching, 2013-2018

Patient Characteristics	Traditional Medicare	Medicare Advantage	SMD
**No. of Person Years**	**25,558,300**	**17,069,188**	
**Age**			
**66-70**	7,725,675 (30.2%)	5,250,849 (30.8%)	0.08
**71-75**	6,420,952 (25.1%)	4,608,082 (27.0%)	
**76-80**	4,565,754 (17.9%)	3,131,128 (18.3%)	
**81-90**	5,464,366 (21.4%)	3,398,408 (19.9%)	
**90+**	1,381,553 (5.4%)	6,807,21 (4.0%)	
**Sex**			
**Male**	10,046,070 (39.3%)	7,024,507 (41.2%)	0.04
**Female**	15,512,230 (60.7%)	10,044,681 (58.8%)	
**Race/Ethnicity**			
**American Indian or Alaskan Native**	87,317 (0.3%)	31,021 (0.2%)	0.29
**Asian**	720,265 (2.8%)	754,234 (4.4%)	
**Black**	1,838,770 (7.2%)	1,776,813 (10.4%)	
**Hispanic**	1,284,142 (5.0%)	1,941,908 (11.4%)	
**Other**	497,813 (1.9%)	340,075 (2.0%)	
**White**	21,129,993 (82.7%)	12,225,137 (71.6%)	
**Dual-eligible status**			
**No**	21,279,055 (83.3)	14,392,889 (84.3)	0.03
**Yes**	4,279,245 (16.7)	2,676,299 (15.7)	
**Low Income Subsidy Eligibility**			
**No**	20,196,895 (79.0%)	13,568,492 (79.5%)	0.01
**Yes**	5,361,405 (21.0%)	3,500,696 (20.5%)	
**Rurality**			
**Not Rural**	20,027,607 (78.4%)	15,430,293 (90.4%)	0.34
**Rural**	5,530,693 (21.6%)	1,638,895 (9.6%)	
**Neighborhood Deprivation**			
**Social Vulnerability Index**	0.40 (10.3)	0.39 (11.7)	0.09

© 2023 Figueroa JF et al. *JAMA Network Open*

eTable 2: HRM Rates in MA vs. TM Beneficiaries Stratified by HRMs Drug Category, 2013-2018

	Differences in the Rate of HRMs per 1,000 Beneficiaries in MA vs. TM (95% CIs)	Example
Anticholinergics, Anti-Parkinson Agents	-0.8 (CI: -0.9, -0.7)	benztropine, trihexyphenidyl
Anticholinergics, 1st Generation Antihistamines	-3.9 (CI: -5.1, -2.8)	chlorpheniramine, diphenhydramine
Antithrombotic	-0.1 (CI: -0.1, -0.1)	dipyridamole, ticlopidine
Cardiovascular, Alpha Agonists, Central	-0.2 (CI: -0.2, -0.1)	guanfacine, methyldopa
Cardiovascular, Other	-0.03 (CI: -0.06, -0.01)	disopyramide, Nifedipine
Central Nervous System, Antidepressants	-1.6 (CI: -1.8, -1.3)	amitriptyline, imipramine
Central Nervous System, Barbiturates	-1 (CI: -1.4, -0.7)	acetaminophen/butalbital/caffeine, Phenobarbital
Central Nervous System, Other	-0.02 (CI: -0.04, -0.01)	meprobamate
Central Nervous System, Vasodilators	-0.004 (CI: -0.007, -0.005)	ergoloid mesylates, isoxsuprine
Endocrine System, Estrogens	-4.2 (CI: -4.9, -3.6)	conjugated estrogens, estradiol
Endocrine System, Other	-2.8 (CI: -3.3, -2.4)	megestrol, thyroid desiccated
Endocrine System, Sulfonylureas, Long-Duration	-1.3 (CI: -1.7, -0.8)	chlorpropamide, glyburide
Pain Medications, Other	-1.4 (CI: -2.1, -0.8)	indomethacin, ketorolac
Pain Medications, Skeletal Muscle Relaxants	-6.9 (CI: -8.5, -5.2)	carisoprodol, chlorzoxazone

eTable 3: HRM Rates Among Medicare Advantage PPO Compared to HMO Plans, 2013-2018

	Differences in MA PPO vs HMO Plans
**Adjusted Rate of HRMs per 1,000 Beneficiaries**	9.4 (CI: 4.4, 14.5)
**Adjusted Proportion of Beneficiaries that Received at least 1 HRMs**	0.8% (CI: 0.4%, 1.2%)

eTable 4: HRM rates in MA vs. TM Beneficiaries After Expanding Study Definition of HRM to Any NDCs Flagged Within a Given Year, 2013-2018

	Primary Outcome	Secondary Outcome
	Differences in the Rate of HRMs per 1,000 Beneficiaries in MA vs. TM	Differences in the Proportion of Beneficiaries that Received at least 1 HRM in MA vs. TM (Percentage Points)
**Main Analysis (flag common NDCs)**	-24.3 (CI: -28.3 to -20.2)	-2.4% (CI: -2.8%, -2.0%)
**Sensitivity Analysis (flag all NDCs)**	-73.4 (CI: -84.1, -62.9)	-5.8% (CI: -6.6%, -5.0%)

© 2023 Figueroa JF et al. *JAMA Network Open*

eTable 5: HRM Rates in MA vs. TM Beneficiaries Using Negative Binomial Regressions and Logistic Regressions, 2013-2018

	**Differences in HRM Use Among MA vs. TM beneficiaries**
**Adjusted Rate of HRMs per 1,000 Beneficiaries** (quasi-Poisson regression)	0.72 (CI: 0.69, 0.76)
**Odds ratio of receipt of at least 1 unique HRMs** (logistic regression)	OR 0.71 (CI: 0.68, 0.75)

eTable 6: Differences in Secondary HEDIS DAE Measures among MA vs. TM beneficiaries, 2013-2018

	**Difference in Proportion of MA Beneficiaries compared to TM (Percentage Point Difference)**
**Receipt of at least 1 HRM** (Main analysis)	-2.4% (CI: -2.8%, -2.0%)
**Receipt of at least 2 unique HRMs**	-3.0% (CI: -3.5%, -2.4%)
**Receipt of 2 or more prescriptions of the same HRM**	-2.7% (CI: -2.8%, -2.5%)

eTable 7: HRM rates in MA vs. TM Beneficiaries that remained alive for all 12 months of a year, 2013-2018

	**Differences in the Rate of HRMs per 1,000 Beneficiaries in MA vs. TM**
**Main Analysis**	-24.3 (CI: -28.3 to -20.2)
**Only beneficiaries alive 12 months of a year**	-24.0 (CI: -28.1, -20.0)

eTable 8: HRM Rates in MA vs. TM Beneficiaries with County rather than HRR Fixed Effects, 2013-2018

	**Differences in the Rate of HRMs per 1,000 Beneficiaries in MA vs. TM**
**Main Analysis (HRR fixed-effects)**	-24.3 (CI: -28.3 to -20.2)
**County fixed-effects Model**	-24.1 (CI: -28.0 to -20.0)

© 2023 Figueroa JF et al. *JAMA Network Open*

eTable 9: HRM Rates in MA vs. TM Beneficiaries Adjusting for Patient HCC Risk Score vs # of non-HRMs Received, 2018

Adjustment for Patient Severity	Differences in the Rate of HRMs per 1,000 Beneficiaries in MA vs. TM
# Non-HRMs Received	-15.3 (CI: -19.3, -11.2)
HCC Risk score	-15.6 (CI: -19.5, -11.7)
HCC Risk score (deflated 6% for MA)	-15.2 (CI: -19.1, -11.3)
HCC Risk score (deflated 11% for MA)	-15 (CI: -18.9, -11.1)
HCC Risk score (deflated 16% for MA)	-14.8 (CI: -18.6, -10.9)

© 2023 Figueroa JF et al. *JAMA Network Open*
