# Supplementary material for: Use of High-Risk Medications Among Older Adults Enrolled in Medicare Advantage Plans vs Traditional Medicare
Source: JAMA Netw Open. 2023 Jun 27;6(6):e2320583. doi: 10.1001/jamanetworkopen.2023.20583 (PMC10300714; doi:10.1001/jamanetworkopen.2023.20583)
Supplement: Supplement 2. — Data Sharing Statement [file jamanetwopen-e2320583-s002.pdf]

Data Sharing Statement

Figueroa. Use of High-Risk Medications Among Older Adults Enrolled in Medicare Advantage Plans vs Traditional Medicare. *JAMA Netw Open*. Published June 27, 2023. doi:10.1001/jamanetworkopen.2023.20583

Data
Data available: No

Additional Information
Explanation for why data not available: Data use agreement with CMS restricts the sharing of research-identifiable Medicare data. However, any researcher can apply for the same data by pursuing their own data use agreement with CMS and ResDAC.
